# Prediction of early bladder outcomes after spinal cord injury: The HALT score

**DOI:** 10.1111/cns.14628

**Published:** 2024-02-07

**Authors:** Xiangbo Wu, Xiao Xi, Mulan Xu, Ming Gao, Ying Liang, Miaoqiao Sun, Xu Hu, Li Mao, Xingkai Liu, Chenguang Zhao, Xiaolong Sun, Hua Yuan

**Affiliations:** ^1^ Department of Rehabilitation Medicine, Xijing Hospital Air Force Medical University (Fourth Military Medical University) Xi'an China; ^2^ Department of Rehabilitation Medicine, Shenshan Medical Center, Sun Yat‐sen Memorial Hospital Sun Yat‐sen University Shanwei Guangdong China; ^3^ Department of Health Statistics Air Force Medical University (Fourth Military Medical University) Xi'an China

**Keywords:** bladder outcome, HALT, H‐reflex, prediction model, spinal cord injury

## Abstract

**Aims:**

Neurogenic bladder (NB) is a prevalent and debilitating consequence of spinal cord injury (SCI). Indeed, the accurate prognostication of early bladder outcomes is crucial for patient counseling, rehabilitation goal setting, and personalized intervention planning.

**Methods:**

A retrospective exploratory analysis was conducted on a cohort of consecutive SCI patients admitted to a rehabilitation facility in China from May 2016 to December 2022. Demographic, clinical, and electrophysiological data were collected within 40 days post‐SCI, with bladder outcomes assessed at 3 months following SCI onset.

**Results:**

The present study enrolled 202 SCI patients with a mean age of 40.3 ± 12.3 years. At 3 months post‐SCI, 79 participants exhibited complete bladder emptying. Least absolute shrinkage and selection operator (LASSO) and multivariate logistic regression analyses identified the H‐reflex of the soleus muscle, the American Spinal Injury Association Lower Extremity Motor Score (ASIA‐LEMS), and the time from lesion to rehabilitation facility (TLRF) as significant independent predictors for bladder emptying. A scoring system named HALT was developed, yielding a strong discriminatory performance with an area under the receiver operating characteristics curve (aROC) of 0.878 (95% CI: 0.823–0.933). A simplified model utilizing only the H‐reflex exhibited excellent discriminatory ability with an aROC of 0.824 (95% CI: 0.766–0.881). Both models demonstrated good calibration via the Hosmer–Lemeshow test and favorable clinical net benefits through decision curve analysis (DCA). In comparison to ASIA‐LEMS, both the HALT score and H‐reflex showed superior predictive accuracy for bladder outcome. Notably, in individuals with incomplete injuries, the HALT score (aROC = 0.973, 95% CI: 0.940–1.000) and the H‐reflex (aROC = 0.888, 95% CI: 0.807–0.970) displayed enhanced performance.

**Conclusion:**

Two reliable models, the HALT score and the H‐reflex, were developed to predict bladder outcomes as early as 3 months after SCI onset. Importantly, this study provides hitherto undocumented evidence regarding the predictive significance of the soleus H‐reflex in relation to bladder outcomes in SCI patients.

## Graphical abstract

We have successfully devised two straightforward and dependable models, namely the HALT score and H‐reflex, for the early prediction of bladder outcomes within 3 months following the initiation of spinal cord injury. Both models exhibited remarkable accuracy and discrimination in their predictive capabilities.

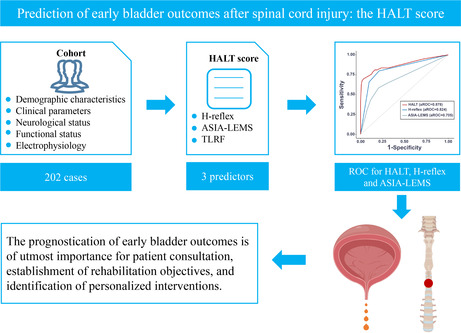

## 1 INTRODUCTION

Spinal cord injury (SCI) represents a devastating event, predominantly affecting individuals aged between 29 and 43, imposing significant burdens on both patient families and society.^1^, ^2^ Neurogenic bladder (NB) has emerged as a prevalent and detrimental consequence of SCI, impacting approximately 80% of SCI patients in the United States^3^ and an alarming 94.65% in Brazil.^4^ NB is characterized by involuntary bladder voiding, increased activity, reduced capacity, and compromised bladder wall compliance due to fibrosis.^5^ There is an increasing consensus suggesting that patients with NB and inadequate bladder management face a heightened risk of urinary tract infection (UTI).^3^, ^6^ UTIs constitute the most common cause of readmission and the second leading cause of death among individuals with SCI.^7^, ^8^

The restoration of voluntary urination function is of paramount importance in the rehabilitation of individuals with SCI, often exceeding the significance of regaining ambulation or alleviating chronic pain.^9^ Bladder management, particularly with the adoption of sterile or clean intermittent catheterization (CIC), has witnessed significant advancements. The primary objective of bladder management is to eliminate the need for CIC once the residual urine volume decreases to less than 100 mL.^10^ Despite numerous interventions, recovery of bladder function following SCI remains limited, with a substantial proportion of individuals (up to 77.3%) unable to achieve voluntary urination and relying on catheterization or CIC for an extended period.^11^ The adoption of CIC introduces inconveniences such as dependence on others and frequent urine leakage, prompting patients to discontinue this practice.^12^ Hence, accurate prediction of bladder outcomes is crucial for patient consultation, rehabilitation goal setting, identification of personalized interventions,^13^ and patient stratification in future clinical trials.^14^

In 2016, the European Multicenter SCI (EMSCI) Study Group proposed the American Spinal Injury Association lower extremity motor score (ASIA‐LEMS) model to predict bladder function outcomes after SCI at 1 year.^15^ This model represented a significant milestone as the first of its kind and demonstrated remarkable predictive proficiency, with an area under the receiver operating characteristics curve (aROC) of 0.912. However, no scoring system has been established within 1 year following SCI. Additionally, the ASIA‐LEMS model heavily relies on subjective physical examination, introducing variability with different examiners. Nerve electrophysiological examination provides an objective and quantitative approach to evaluating nerve function.^16^ The electrically induced bulbocavernosus reflex (E‐BCR), evaluated through the stimulation of the dorsal nerve of the penis or clitoris and recording the bulbocavernosus muscles, offers valuable information regarding the integrity of sacral spinal segments 2 (S2) to 4 (S4), critical for urination control.^17^ However, the invasive nature of E‐BCR may cause discomfort, potentially leading to patient reluctance to cooperate and posing challenges in the clinical prediction of bladder outcomes.^18^ On the other hand, the H‐reflex of the soleus muscle, commonly employed to evaluate the neurological function of the S1 nerve root and its proximal medullary segment,^19^, ^20^ is a simpler and well‐tolerated procedure. Given the anatomical continuity of S1 with segments S2 to S4,^21^ it is hypothesized that the soleus H‐reflex may serve as a potential indicator for predicting bladder function in SCI patients. To address this, a retrospective study was conducted to analyze demographic data, clinical features, and electrophysiological data. The objective was to establish and validate a predictive model for bladder outcomes at 3 months following the onset of SCI, utilizing the H‐reflex of the soleus muscle as an indicator for evaluating the neurological function of the S1 nerve root and its proximal medullary segment.

## 2 METHODS

### 2.1 Study design and patient population

The data utilized in this retrospective study were gathered from all eligible SCI inpatients at the Department of Rehabilitation Medicine, Xijing Hospital, from May 2016 to December 2022, with a disease duration of less than 40 days.^22^ As per the study protocol, all predictor variable data were obtained within 40 days of the onset of the condition, while bladder outcome data were acquired from patients 3 months after the injury. The inclusion criteria were as follows: (1) aged 18 to 79 years; (2) adhering to the diagnostic criteria for SCI as outlined in the International Standard for Neurological Classification^23^; (3) having bladder emptying difficulties and using an indwelling catheter or CIC for emptying^24^; (4) after spinal shock recovery clinically^25^; and (5) comprehensive medical records are available, including documentation of the bladder's condition at 3‐month post‐injury. Individuals with lumbar disc herniation, peripheral neuropathy, diabetes mellitus, or any other conditions that could potentially impact the soleus H‐reflex pathways were excluded from the study.^17^ Data were collected by trained rehabilitation physicians and therapists using a standardized case report form.

This study was conducted in accordance with the Declaration of Helsinki, ensuring patients' rights, and received approval from the Ethics Committee of Xijing Hospital (KY20222096‐C‐1). Furthermore, the study was registered in the Chinese Clinical Trial Registry (ChiCTR; ChiCTR2300073824). As a retrospective and non‐invasive study, the necessity for informed consent was deemed unnecessary. The bladder management implemented in this study was tailored to each patient and followed the Guidelines on Adult Neurogenic Lower Urinary Tract Dysfunction.^26^

### 2.2 Definition of candidate predictor variables

In this section, we delineate the key patient characteristics and variables associated with SCI that serve as candidate predictor variables for our study. Two variables related to key patient characteristics were considered: (1) age and (2) gender.

For SCI‐related variables, three aspects were taken into account: (1) surgery, coded as yes or no^27^; and (2) SCI type, coded as traumatic or non‐traumatic. Non‐traumatic SCI is defined as injury caused by ischemia, spinal stenosis, tumor, transverse myelitis, and infection^28^, ^29^; (3) time from lesion to rehabilitation facility (TLRF) is defined as the number of days between injury and inpatient rehabilitation.^27^ For non‐traumatic lesions, it is essential to clarify that the onset of the lesion is determined by the actual initiation of factors such as inflammation or vascular disease, not merely the date of diagnosis, as indicated by previous research.^30^ A seamless transition into the rehabilitation phase is crucial for optimal patient care. To achieve this, a comprehensive evaluation by an experienced physician is imperative. This evaluation encompasses a thorough assessment of vital signs, injury etiology and mechanisms, physical examination, and ancillary tests.^12^ The receiver operating characteristics (ROC) curve was constructed to evaluate the efficacy of TLRF in predicting bladder outcomes 3 months after SCI. The threshold with the Youden index (sensitivity + specificity – 1) was identified as the optimal cutoff point.^31^ In the present study, the Youden index reached its peak value at 25 days, leading to the adoption of this specific threshold for categorizing TLRF durations as exceeding or falling below 25 days.

Our study integrates seven variables related to the neurological status, aligning with the International Standards for Neurological Classification of Spinal Cord Injury (ISNCSCI): (1) neurological level: as the primary center of urination is in the sacral cord (S2–S4),^32^ the neurological level was divided into S1–S5 and C1–L5; (2) the American Spinal Injury Association (ASIA) Impairment Scale (AIS) grades of severity have been classified as five different grades (A–E), with A indicating a complete lesion and E indicating normal sensation and motor function in all segments^22^; (3) ASIA‐LEMS is the sum of the strength of five pairs of key muscles of the lower limbs (hip flexors, knee extensors, ankle dorsi extensors, digitorum longus, and ankle plantar flexors) assessed by the manual muscle testing (MMT) method.^22^ The total score is 50 points, which is divided into six categories during the analysis: 0, 1–10, 11–20, 21–30, 31–40, and 41–50; (4) S4–S5 dermatome sensation was categorized as absent, impaired, and normal; (5) deep anal pressure was categorized as absent or present; (6) anal reflex was categorized as absent or present; and (7) voluntary anal contraction was categorized as absent or present.^33^

Two variables were derived from the functional status upon admission for rehabilitation: (1) The Modified Barthel Index (MBI) measures the functional status of the patient's activities of daily living. The individual score is determined by various independent behavioral measures, and the total score ranges from 0 to 100 points^33^; and (2) The Spinal Cord Independence Measure (SCIM) is specifically crafted to evaluate the functional capacity of SCI patients and assess their ability to perform SCI‐related activities of daily living. The total SCIM score ranges from 0 to 100, with higher scores indicating greater independence.^33^

One parameter under consideration was urinary tract infection as a complication. UTI was characterized by a bacteriuria count equal to or exceeding 10^5^ colony‐forming units per milliliter (CFU/mL), along with the concurrent presence of at least one acute clinical sign of infection occurring within 48 h (e.g., chills, fever, sweating, and cloudy/malodorous urine).^7^, ^25^, ^34^

The H‐reflex of the soleus muscle was also included as a candidate variable. Following participant enrollment, the soleus H‐reflex was measured using MEB‐9404C electromyography. The patient assumed a prone position with legs in a naturally extended and fully relaxed state. The recording electrode was positioned on the soleus muscle, while the reference electrode was placed on the Achilles tendon. Stimulation of the tibial nerve occurred at the popliteal fossa, with the cathode of the stimulation electrode facing proximally. The intensity of stimulation ranged from 0 to 50 mA. The H‐reflex appeared at about 25 to 30 ms.^35^ The amplitude and latency of the left and right H‐waves were recorded. To facilitate the use of electrophysiological tests for predictive models, the H‐reflex in this study was divided into three categories: (1) the H‐reflex (+) group: H‐reflex was present on both sides; (2) the H‐reflex (±) group: H‐reflex was present on one side; and (3) the H‐reflex (−) group: H‐reflex was absent on both sides.^17^, ^35^

### 2.3 Outcome measure

The outcome of the study was complete bladder emptying in patients after SCI at 3 months, defined as a residual urine volume of less than 100 mL on three consecutive occasions, as assessed through ultrasound or catheterization.^36^, ^37^ Patients were dichotomized based on bladder function: those demonstrating complete bladder emptying and those who did not.

### 2.4 Statistical analysis

The normality of the data was assessed using the Shapiro–Wilk test. Continuous variables were presented as mean ± standard deviation for those with a normal distribution and as median and interquartile range for those with a non‐normal distribution. Categorical variables were analyzed using the Chi‐square test, and continuous variables were evaluated using either the Mann–Whitney U test or Student's *t*‐test. To address potential model instability arising from a limited sample size, variable selection employed the least absolute shrinkage and selection operator (LASSO) regression.^38^ Following this, a multiple logistic regression analysis was performed to develop a predictive model.^39^ Each independent risk factor received scores by dividing its beta coefficient in the model by the lowest beta coefficient, rounding to the nearest integer. The prediction score was derived by summing the scores for each factor, with higher scores indicating a greater likelihood of complete bladder emptying in SCI patients.^40^ The bootstrap method was employed to sample 1000 times and calculate the aROC to validate the predictive efficacy of the prediction score.^41^ An aROC greater than 0.75 was considered indicative of relatively excellent predictive performance.^42^ Calibration assessment involved the calibration curve, Hosmer–Lemeshow test, and Brier scores. The Brier score, considering both calibration and discrimination, measured the mean‐squared difference between observed and predicted outcomes, ranging from 0.0 (perfect prediction) to 0.25 (poor prediction) in cases with a 50% outcome incidence.^43^ Clinical validity was examined through decision curve analysis (DCA), and a nomogram was developed based on statistically significant predictors. Statistical analyses were performed using SPSS 24.0 and R 4.0, adhering to Strengthening the Reporting of Observational Studies in Epidemiology (STROBE) guidelines (Data S1). A *p*‐value <0.05 was statistically significant.

## 3 RESULTS

### 3.1 Patients' characteristics

**FIGURE 1:**
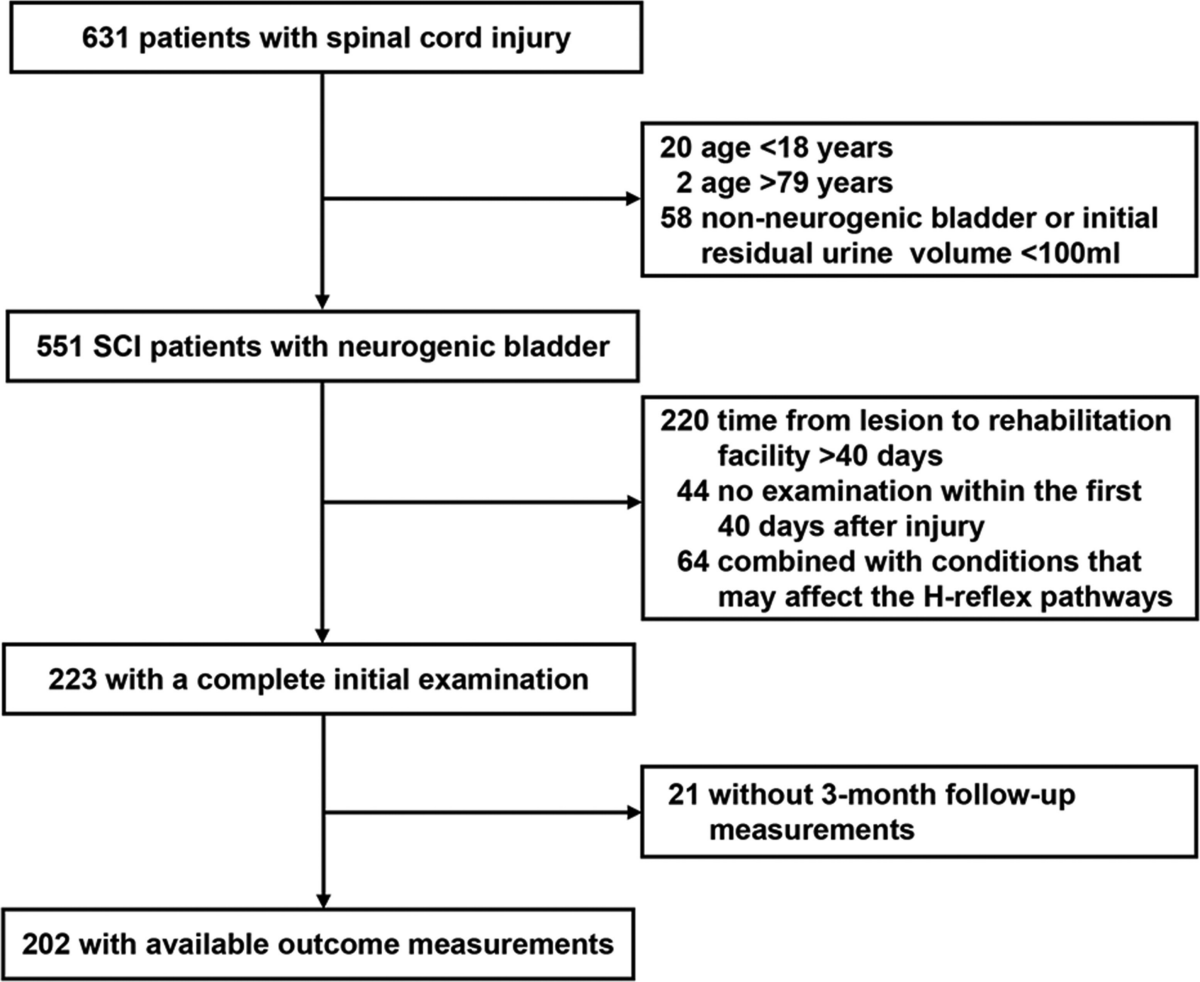
Selection of patients.

A total of 631 hospitalized patients with SCI were initially considered. Following adherence to inclusion and exclusion criteria, 202 patients were eligible for the final analysis (Figure 1). The clinical characteristics of the analyzed patients were comparable to those lost to follow‐up (Table S1). Based on bladder outcomes at the 3‐month mark, the 202 patients were categorized into the complete bladder emptying group (*n* = 79) and the incomplete bladder emptying group (*n* = 123). Demographic characteristics, clinical parameters, neurological status, functional status, complications, and electrophysiology of these two groups are detailed in Table 1.

**TABLE 1:** Characteristics of the subjects who achieved or did not achieve complete bladder control.

Variable	Incomplete bladder emptying group (*n* = 123**)**	Complete bladder emptying group (*n* = 79**)**	*p*‐value
Demographic characteristics			
Age, mean ± SD, years	39.6 ± 11.6	41.0 ± 13.9	0.466 (Student's *t*‐test)
Gender			
Male	99 (80.5%)	60 (75.9%)	0.442 (*χ* ^2^)
Female	24 (19.5%)	19 (24.1%)	
Clinical parameters			
Surgery			
Yes	120 (97.6%)	72 (91.1%)	**0.040** (*χ* ^2^)
No	3 (2.4%)	7 (8.9%)	
Etiology			
Non‐traumatic	8 (6.5%)	13 (16.5%)	**0.024** (*χ* ^2^)
Traumatic	115 (93.5%)	66 (83.5%)	
TLRF, median (IQR), days	30.0 (11.0, 36.0)	16.0 (9.0, 30.0)	**0.002** (Mann–Whitney U)
Neurological status			
Neurological Level			
C1–L5	118 (95.9%)	75 (94.9%)	0.737 (*χ* ^2^)
S1–S5	5 (4.1%)	4 (5.1%)	
Severity of initial neurological deficit			
AIS grade A	96 (78.0%)	33 (41.8%)	**<0.001** (*χ* ^2^)
AIS grade B	15 (12.2%)	15 (19.0%)	
AIS grade C	6 (4.9%)	16 (20.2%)	
AIS grade D	6 (4.9%)	15 (19.0%)	
AIS grade E	0 (0%)	0 (0%)	
ASIA‐LEMS, median (IQR)	0 (0, 6.0)	15.0 (0, 36.0)	**<0.001** (Mann–Whitney U)
S4–S5 dermatome sensation			
Normal	59 (48.0%)	26 (32.9%)	**0.007** (*χ* ^2^)
Impaired	17 (13.8%)	25 (31.6%)	
Absent	47 (38.2%)	28 (35.5%)	
Deep anal pressure			
Present	74 (60.1%)	46 (58.2%)	0.785 (*χ* ^2^)
Absent	49 (39.9%)	33 (41.8%)	
Anal reflex			
Present	73 (59.3%)	48 (60.7%)	0.842 (*χ* ^2^)
Absent	50 (40.7%)	31 (39.3%)	
Voluntary anal contraction			
Present	72 (58.5%)	46 (58.2%)	0.965 (*χ* ^2^)
Absent	51 (41.5%)	33 (41.8%)	
Functional status			
MBI, median (IQR)	13.0 (5.0, 26.0)	9.0 (3.0, 25.0)	0.210 (Mann–Whitney U)
SCIM total score, median (IQR)	18.0 (12.0, 27.0)	15.0 (10.0, 30.0)	0.400 (Mann–Whitney U)
Complications			
Urinary tract infection			
Yes	36 (29.3%)	16 (20.3%)	0.153 (*χ* ^2^)
No	87 (70.7%)	63 (79.7%)	
Electrophysiology			
H‐reflex			
H‐reflex (−)	97 (78.9%)	16 (20.3%)	**<0.001** (*χ* ^2^)
H‐reflex (±)	14 (11.4%)	11 (13.9%)	
H‐reflex (+)	12 (9.8%)	52 (65.8%)	

Abbreviations: AIS, American Spinal Injury Association Impairment Scale; ASIA‐LEMS, the American Spinal Injury Association lower extremity motor score; IQR, interquartile range; MBI, the Modified Barthel Index; SCIM, the spinal cord independence measure; SD, standard deviation; TLRF, time from lesion to rehabilitation facility.The bold indicate significance values.

### 3.2 Development of an Individualized Prediction Model

**FIGURE 2:**
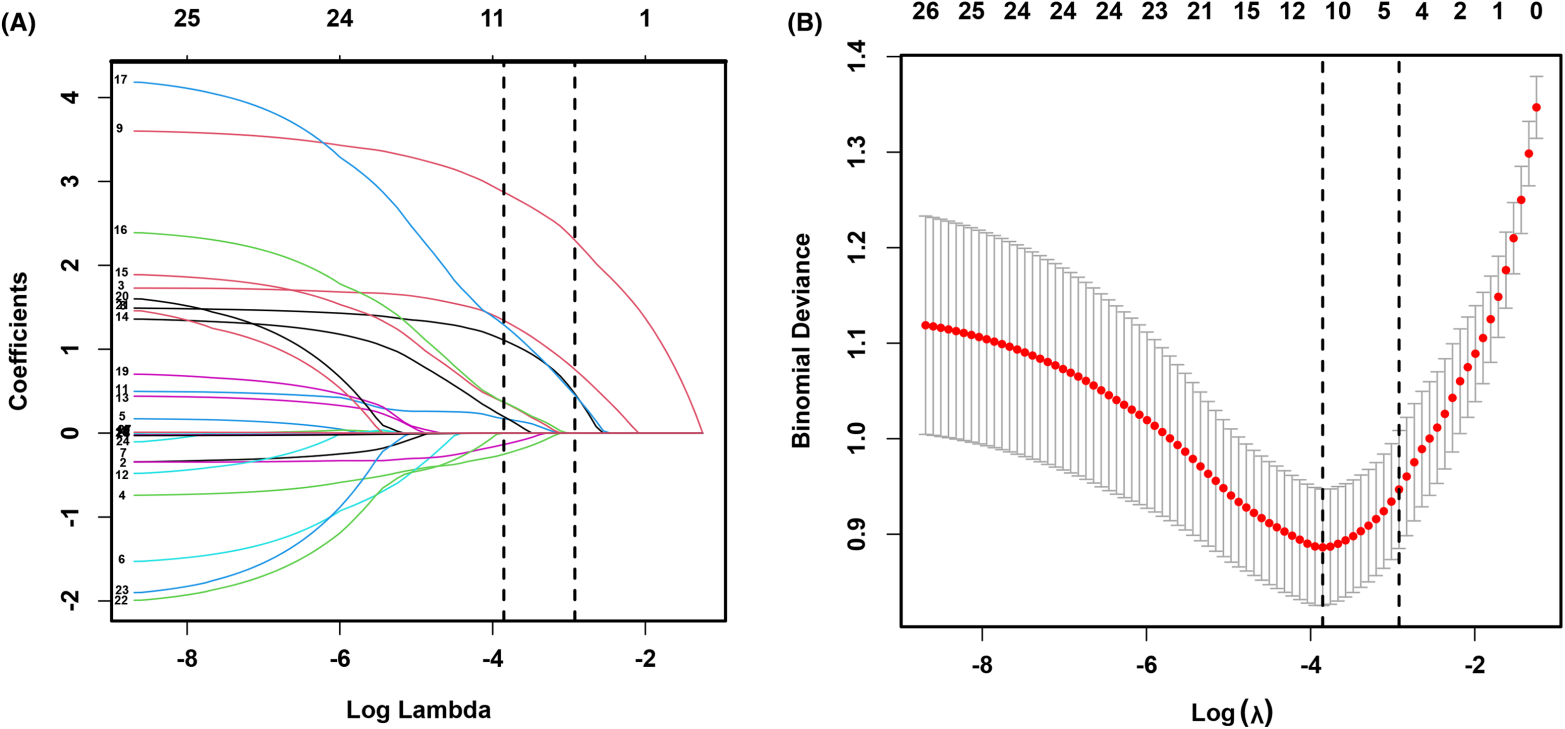
Features selection using the LASSO regression model. (A) Log Lambda value of the 16 features in the LASSO model. A coefficient profile plot was produced against the log lambda sequence; and (B) parameter selection in the LASSO model used 10‐fold cross‐validation via minimum criterion. The binomial deviance was computed for the test data as measures of the predictive performance of the fitted models. The built‐in function in R produces two automatic λs—one that minimizes the binomial deviance and one representing the largest λ that is still within 1 standard error of the minimum binomial deviance. Abbreviations: LASSO, least absolute shrinkage and selection operator.

From the pool of 16 candidate predictor variables, H‐reflex, ASIA‐LEMS, and TLRF were selected based on non‐zero coefficients determined by LASSO logistic regression (Figure 2). These variables were subsequently incorporated into multivariate logistic regression analysis, identifying H‐reflex, ASIA‐LEMS, and TLRF as independent predictors of autonomous bladder emptying (Table 2). For practical clinical application, a scoring system named HALT (acronym for H‐reflex, ASIA‐LEMS, and TLRF) was devised (Table 3), with a total score for each patient obtained by summing the points for each predictor variable on a scale from 0 to 24. Higher scores indicated a higher likelihood of complete bladder emptying at 3 months (Table S2).

**TABLE 2:** Multivariate logistic regression analysis of factors within 3 months.

Variable	*β*	Wald	*p*‐value	Odds ratio (95% CI)
H‐reflex				
H‐reflex (−)	—	—	—	—
H‐reflex (±)	1.544	6.421	**0.011**	4.685 (1.419 to 15.467)
H‐reflex (+)	3.582	45.047	**<0.001**	35.928 (12.625 to 102.25)
ASIA‐LEMS				
0	—	—	—	—
1–10	0.315	0.149	0.700	1.371 (0.276 to 6.806)
11–20	1.017	2.183	0.140	2.765 (0.717 to 10.661)
21–30	1.143	2.426	0.119	3.136 (0.744 to 13.216)
31–40	1.198	1.829	0.176	3.315 (0.584 to 18.828)
41–50	2.346	9.397	**0.002**	10.448 (2.331 to 46.837)
TLRF, days				
>25	—	—	—	—
≤25	1.994	17.439	**<0.001**	7.342 (2.880 to 18.713)

Abbreviations: ASIA‐LEMS, the American Spinal Injury Association lower extremity motor score; TLRF, time from lesion to rehabilitation facility.The bold indicate significance values.

**TABLE 3:** Point allocation for the total score based on regression coefficients.

Relative factor	Categories	Points
H‐reflex	H‐reflex (−)	0
	H‐reflex (±)	5
	H‐reflex (+)	11
ASIA‐LEMS	0	0
	1–10	1
	11–20	3
	21–40	4
	41–50	7
TLRF, days	>25	0
	≤25	6

Abbreviations: ASIA‐LEMS, the American Spinal Injury Association lower extremity motor score; TLRF, time from lesion to rehabilitation facility.

### 3.3 Performance and validation of the HALT score and H‐reflex

**FIGURE 3:**
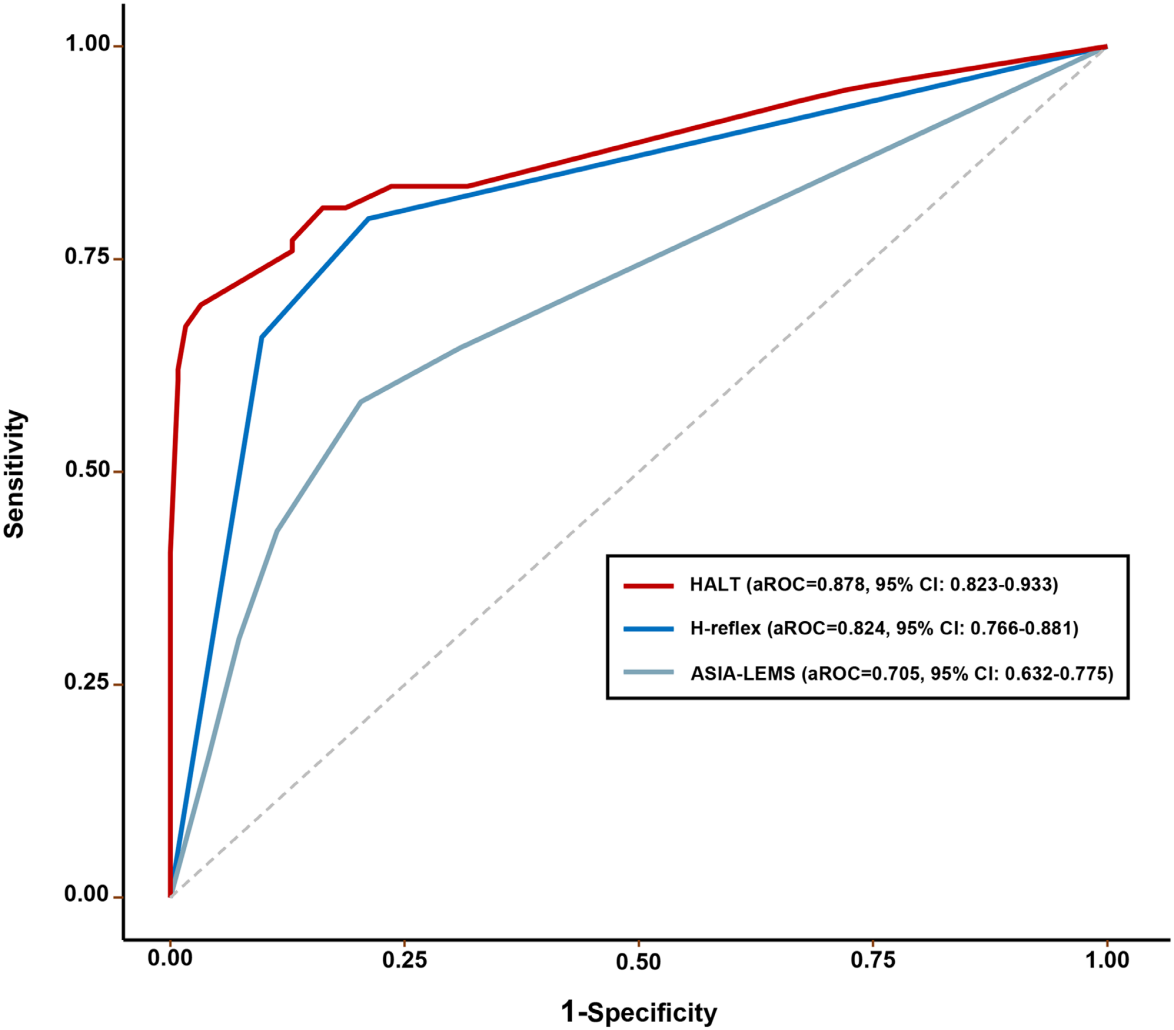
Receiver operating characteristic (ROC) curve for HALT, H‐reflex, and ASIA‐LEMS. HALT: aROC = 0.878, 95% CI: 0.823–0.933; H‐reflex: aROC = 0.824, 95% CI: 0.766–0.881; ASIA‐LEMS: aROC = 0.705, 95% CI: 0.632–0.775. Abbreviations: aROC, area under the receiver operating characteristics curve; ASIA‐LEMS, the American Spinal Injury Association lower extremity motor score; HALT, H‐reflex, ASIA‐LEMS, and TLRF; TLRF, time from lesion to rehabilitation facility.

**FIGURE 4:**
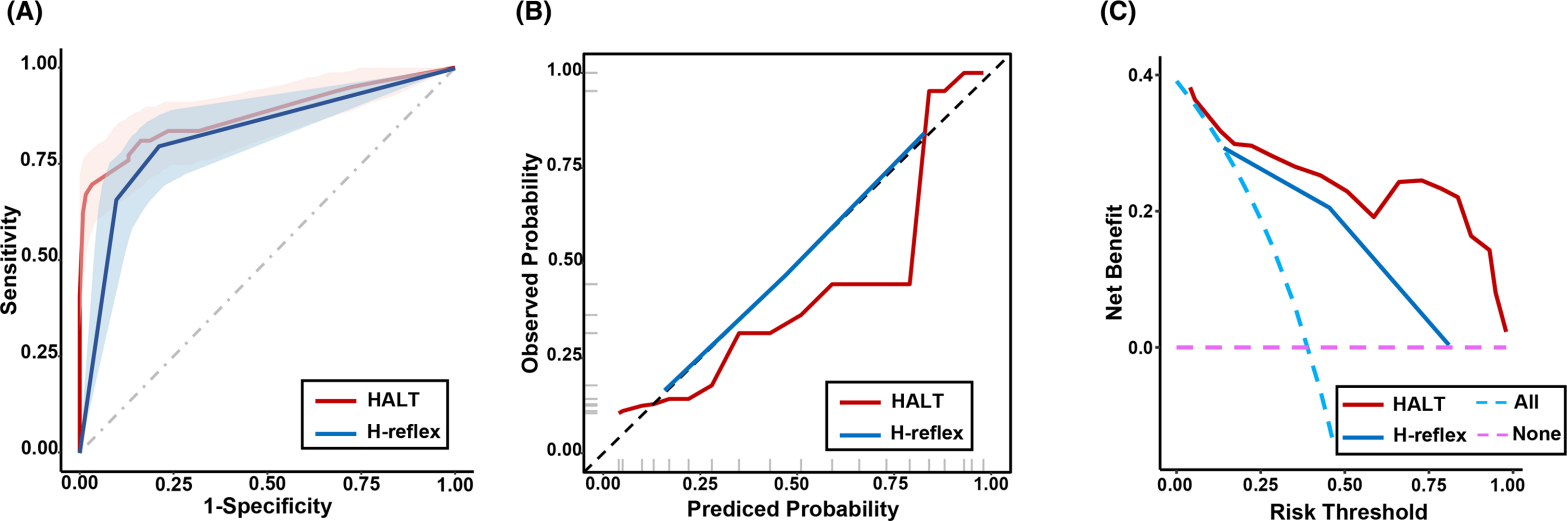
Evaluation of the models for the HALT score and the H‐reflex. (A) Internal validation of the HALT score and the H‐reflex. Receiver operating characteristic (ROC) curve for the HALT score and the H‐reflex generated using bootstrap resampling (1000 times); (B) calibration plot of actual probability versus predicted probability of complete bladder emptying according to the HALT score and the H‐reflex; and (C) decision curve analysis (DCA) for the HALT score and the H‐reflex. The graph depicts the expected net benefit per patient relative to the HALT and H‐reflex of bladder autonomic emptying. The net benefit increases as the model curve is extended. Abbreviations: aROC, area under the receiver operating characteristics curve; ASIA‐LEMS, the American Spinal Injury Association lower extremity motor score; DCA, decision curve analysis; HALT: H‐reflex, ASIA‐LEMS, and TLRF; TLRF, time from lesion to rehabilitation facility.

**FIGURE 5:**
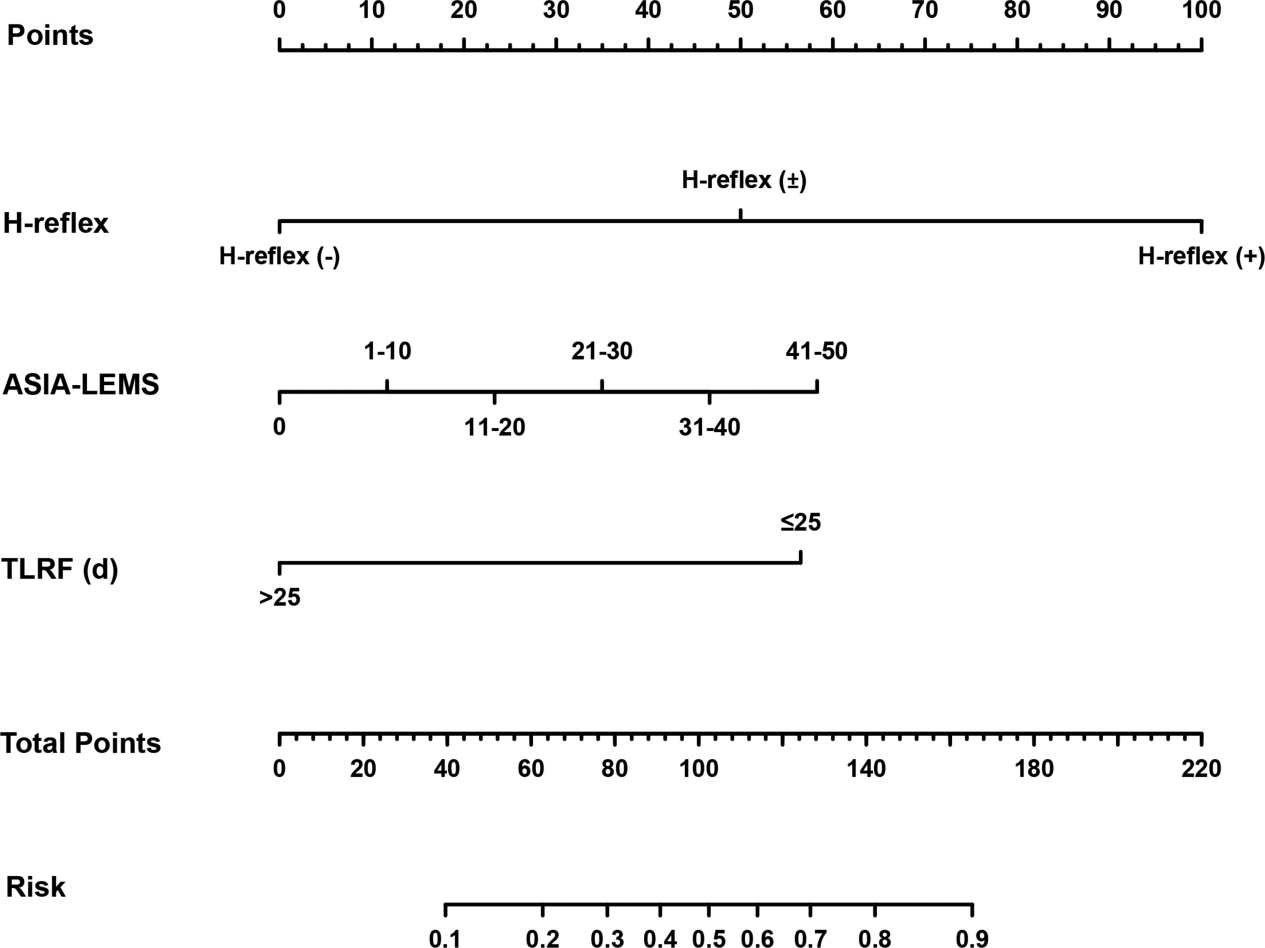
Development of the nomograms. Abbreviations: ASIA‐LEMS, the American Spinal Injury Association lower extremity motor score; TLRF, time from lesion to rehabilitation facility.

The ROC curve demonstrated that the HALT score exhibited good discriminative power with an aROC of 0.878 (95% CI: 0.823–0.933). The aROC for the H‐reflex was 0.824 (95% CI: 0.766–0.881), and for ASIA‐LEMS, it was 0.705 (95% CI: 0.632–0.775; Figure 3). Pairwise comparisons revealed that the aROC of HALT was higher than that of H‐reflex (*p* = 0.001) and ASIA‐LEMS (*p* < 0.001), and the aROC of H‐reflex was higher than that of ASIA‐LEMS (*p* = 0.002). H‐reflex was then defined as the simple model. Internal validation through the bootstrap self‐sampling method (1000 repeated samplings) demonstrated excellent discrimination for both HALT and H‐reflex (Figure 4A). Calibration curve analysis for the HALT model exhibited satisfactory consistency between predicted and reference probability curves, with the Hosmer–Lemeshow test yielding non‐significant results (*p* = 0.106), indicating high consistency (Figure 4B). The Brier score of 0.118 further supported the model's excellent calibration. Similarly, the H‐reflex model showed a robust fit with a non‐significant Hosmer–Lemeshow test (Figure 4B) and a Brier score of 0.147. DCA indicated threshold probabilities for HALT and H‐reflex, ranging from 0.04 to 0.98 and 0.14 to 0.81, respectively, signifying their effectiveness, safety, reliability, and practicality within this range (Figure 4C). To enhance visualization and streamline clinical application, a nomogram for the HALT scoring system was developed (Figure 5).

### 3.4 Subgroup analysis

**FIGURE 6:**
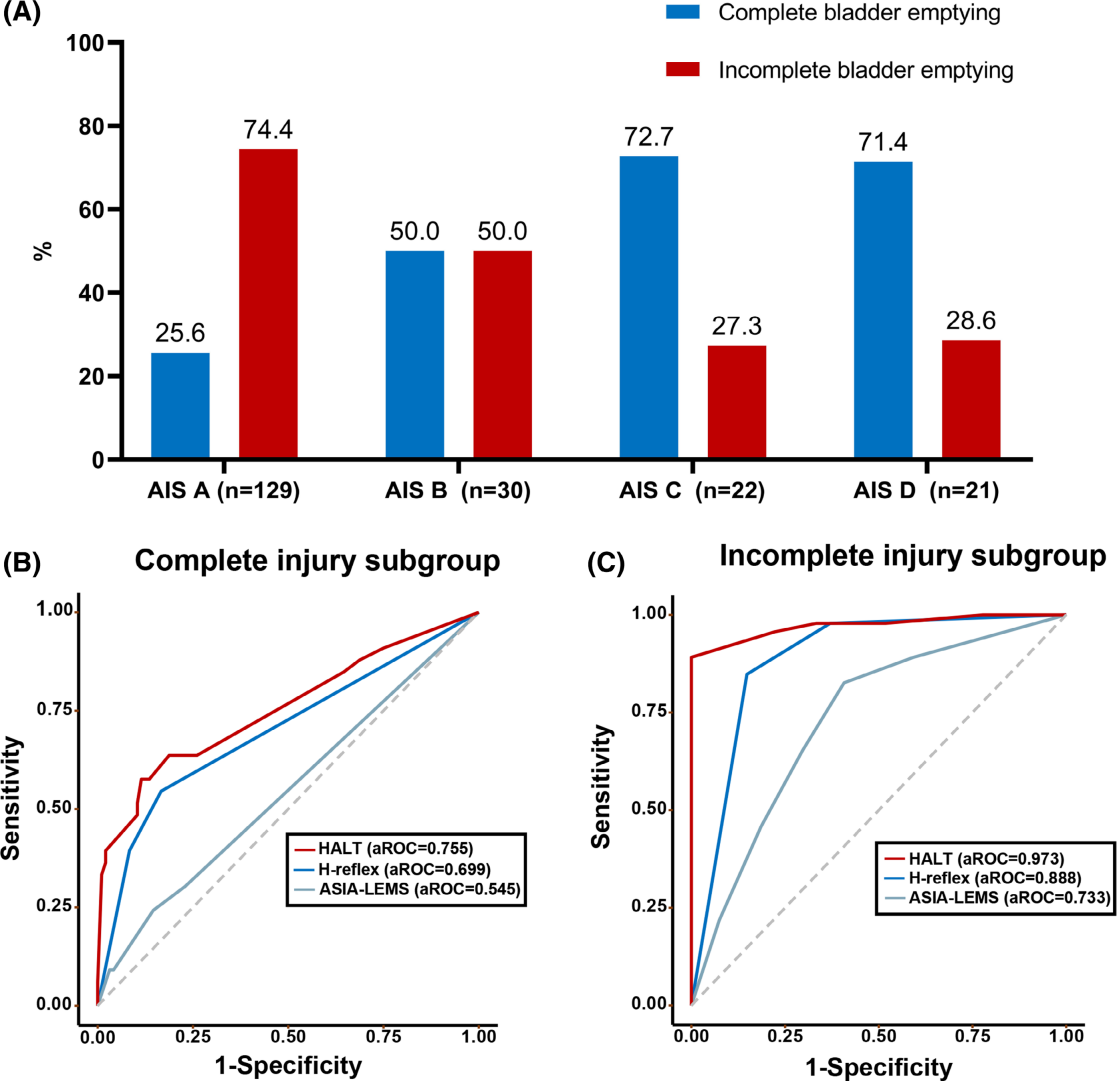
Subgroup analysis by AIS classification. (A) Rates of complete bladder emptying and incomplete bladder emptying at 3‐month follow‐up by AIS classification; (B) receiver operating characteristic (ROC) curve for HALT, H‐reflex, and ASIA‐LEMS in the complete injury subgroup. HALT: aROC = 0.755, 95% CI: 0.648–0.861; H‐reflex: aROC = 0.699, 95% CI: 0.603–0.796; ASIA‐LEMS: aROC = 0.545, 95% CI: 0.451–0.638; (C) receiver operating characteristic curve for HALT, H‐reflex, and ASIA‐LEMS in the incomplete injury subgroup. HALT: aROC = 0.973, 95% CI: 0.940–1.000; H‐reflex: aROC = 0.888, 95% CI: 0.807–0.970; ASIA‐LEMS: aROC = 0.733, 95% CI: 0.611–0.856. Abbreviations: AIS, American Spinal Injury Association impairment scale; aROC, area under the receiver operating characteristics curve; ASIA‐LEMS, the American Spinal Injury Association lower extremity motor score; HALT, H‐reflex, ASIA‐LEMS, and TLRF; TLRF, time from lesion to rehabilitation facility.

At the 3‐month follow‐up, variations in bladder emptying rates were observed across AIS categories, with rates of 25.6% for AIS A, 50.0% for AIS B, 72.7% for AIS C, and 71.4% for AIS D (Figure 6A). Consequently, prediction models were separately applied to patients within the complete injury subgroup (AIS A) and incomplete injury subgroup (AIS B, AIS C, and AIS D). The outcomes indicated a significant decline in the predictive accuracies of HALT (aROC = 0.755, 95% CI: 0.648–0.861) and H‐reflex (aROC = 0.699, 95% CI: 0.603–0.796) in the subgroup of patients with complete injuries (see Figure 6B). Conversely, the predictive accuracies of HALT (aROC = 0.973, 95% CI: 0.940–1.000) and H‐reflex (aROC = 0.888, 95% CI: 0.807–0.970) significantly increased in the subgroup of patients with incomplete injuries (Figure 6C). Statistical analysis confirmed the statistical significance (*p* < 0.05) of these differences in predicted accuracies when compared to the accuracies observed in the entire study population. These findings underscore that HALT and H‐reflex are more suitable for individuals with incomplete spinal cord injuries.

## 4 DISCUSSION

This study introduces the HALT prognostic scoring system to predict bladder function outcomes at the 3‐month mark in patients with SCI. We found that the H‐reflex of the soleus muscle, ASIA‐LEMS, and TLRF were significant predictors of bladder outcomes. Our findings suggest that the HALT scale, comprising 24 points, has huge prospects for seamless integration into clinical practice, offering early insights into bladder function prognosis (aROC = 0.878, 95% CI: 0.823–0.933). Additionally, the study highlights the novel predictive capacity of the soleus H‐reflex in facilitating complete bladder emptying among SCI patients at 3 months, with an aROC of 0.824 (95% CI: 0.766–0.881).

It is now understood that the process of voiding relies on the synchronization of impulses among the cerebral cortex, pontine, and sacral micturition centers. SCI disrupts the neural pathways connecting the sacral cord and superior micturition centers, leading to neurogenic bladder dysfunction.^23^, ^30^ Assessing the neurological functioning of sacral micturition centers involves evaluating sensory and motor functions associated with S2–S4.^44^ Previous studies have investigated the predictive values of sensory and motor examinations on bladder function 6 months or 1 year after SCI based on small samples, yielding conflicting results. In a study by Schurch et al.,^45^ 55 patients with thoracolumbar fractures and SCI were examined to predict bladder function at least 6 months post‐injury. Their findings suggested that sensory assessment was not effective in predicting neurogenic voiding dysfunction. Similarly, Weiss et al.^46^ examined 19 SCI patients and found that perianal pinprick and toe position sense, assessed within 72 h of injury, were highly sensitive predictors of bladder function recovery at 1 year. Specifically, 70% of patients with preserved pinprick sensation and 75% with preserved position sense could voluntarily empty their bladders 1 year after the injury. Shenot et al. studied 28 SCI patients and discovered that within 72 h post‐SCI, intrinsic perineal pin sensing (PPS) predicted 65% of patients who could urinate autonomously at 1 year, and the presence of bulbocavernosus reflex (BCR) predicted 60% of patients.^47^ Recently, Pavese et al., based on a large sample study of EMSCI, developed two predictive models for bladder outcomes at 1 year of post‐traumatic SCI. The comprehensive model included ASIA‐LEMS, light touch at the S3 segment, and SCIM subscale for respiration and sphincter management, while the simplified model solely relied on ASIA‐LEMS. Both models demonstrated exceptional predictive abilities.^15^ The heterogeneity in previous studies may be attributed to the subjective and variable nature of physical examinations, influenced by standardized procedures, examiner expertise, and patient cooperation. It is crucial to note that no scoring system has been developed for assessing individuals with spinal cord injuries within the first year post‐injury.

Electrophysiological techniques have emerged as a promising method for predicting outcomes, offering inherent objectivity, especially in situations where patient cooperation is limited. Hupp et al.^16^ conducted a study demonstrating the effectiveness of electrophysiological multimodal assessments, such as motor evoked potentials, somatosensory evoked potentials, and nerve conduction studies, in accurately predicting functional recovery within 6 to 12 months following traumatic SCI. Specifically, E‐BCR, elicited through stimulation of dorsal and efferent nerves of the penis or clitoris using the sacral reflex arc S2 to S4,^48^ emerged as a potential indicator of bladder function.^49^ In the study by Cha et al.,^50^ E‐BCR exhibited high specificity (88.5%) in predicting bladder outcomes in 106 patients with tethered cord syndrome (TCS) after surgery releasing the tethered cord at 6 months. Lee et al.^17^ demonstrated the advantage of E‐BCR in predicting the prognosis of bladder dysfunction in 40 patients with cauda equina syndrome, showing a higher negative predictive value compared to manually assessed BCR. However, it is important to note that the invasive nature of E‐BCR testing has the potential to induce patient discomfort, leading to significant reluctance among a considerable number of patients to undergo the procedure.^17^ Therefore, the clinical utilization of E‐BCR for prognosticating bladder outcomes in SCI patients' injuries presents notable challenges.^18^

Utilizing LASSO and multiple logistic regression analyses, our study provided hitherto undocumented evidence that patients exhibiting a positive H‐reflex were more likely to achieve complete bladder emptying at 3 months compared to those with H‐reflex (±) or H‐reflex (−). The model demonstrated excellent predictive performance with an aROC of 0.824 (95% CI: 0.766–0.881). The H‐reflex, also known as the Hoffman reflex, represents a monosynaptic spinal cord reflex elicited through electrical stimulation.^51^ This reflex serves as a valuable tool for assessing the excitability of α‐motor neurons in the anterior horn of the spinal cord and the functional integrity of sensory and motor nerve fibers along the conduction pathway.^20^ The soleus H‐reflex provides a non‐invasive method for assessing the proximal segment of peripheral nerves, achieved by stimulating a mixed peripheral nerve like the tibial nerve.^52^ Its user‐friendly nature and consistent results make it detectable in 97.3% of individuals with normal neurological function.^51^ Originating from the soleus muscle, this reflex offers a comprehensive evaluation of the functional state of the S1 nerve root.^53^ The sacral segment, notably indivisible when compared to other spinal cord segments, has been reported to be intact when the soleus H‐reflex is present, indicating preservation of sacral medullary segments' integrity and connections to afferent and efferent pathways in the genitourinary region. Conversely, the absence of the soleus H‐reflex suggests severe damage to the sacral radicular nerve.^54^ Zariffa et al. established a close relationship between the functional preservation of different sacral segments. Their findings accurately predicted (90.5%) sensory preservation of the sacral coccygeal region (S4–S5 sensation) based on sensory preservation of S1. Preserved S1 sensory and motor function predicted distal sacral root function (S4–S5), specifically anal sensation and voluntary contraction.^55^ The tibial nerve, a component of the sciatic nerve originating from the lumbosacral plexus (L4 to S3 segments), gives rise to nerve branches innervating the bladder and pelvic floor.^56^ Stimulating the tibial nerve, such as through percutaneous tibial nerve electrical stimulation (PTNS), can depolarize afferent nerves in these segments, playing a significant role in neurogenic bladder management.^52^ Considering its convenience and patient acceptance, the soleus H‐reflex represents a more suitable predictor of bladder function among individuals with SCI in clinical settings compared to E‐BCR. However, it should be borne in mind that the H‐reflex may be abnormal in patients with S1 compression from lumbar disc herniation, necessitating their exclusion from the predictive model.

It is widely acknowledged that ASIA‐LEMS involves the manual muscle testing of five key muscles in the lower limbs, corresponding to the L2–S1 spinal cord segment.^22^ Pavese et al. initially discovered that ASIA‐LEMS exhibited remarkable predictive efficacy in forecasting complete bladder emptying 1 year following traumatic SCI, as evidenced by the EMSCI database. This exceptional predictive ability was externally validated,^15^, ^57^ and ASIA‐LEMS also demonstrated outstanding predictive ability for complete bladder emptying 1 year after ischemic SCI using the same database. Importantly, ASIA‐LEMS is characterized by its simplicity, expediency, non‐invasiveness, affordability, and lack of specialized equipment requirements.^13^ Elliott et al.^57^ further validated the predicted efficacy of the ASIA‐LEMS model in bladder outcomes 1 year after SCI using the National Spinal Cord Injury Database (NSCID). However, our study found that ASIA‐LEMS had a moderate performance in predicting spontaneous bladder emptying in patients after SCI at 3 months (aROC = 0.705, 95% CI: 0.632–0.775), significantly poorer than H‐reflex (aROC = 0.826, 95% CI: 0.766–0.881, *p* = 0.002). The occurrence of a secondary phase of SCI following the initial trauma is well‐documented, involving factors like ischemia, excitotoxicity, neuroinflammation, and oxidative stress. Notably, this secondary SCI may manifest several days or weeks after the initial injury.^58^ Relying solely on physical examination, as in the commonly used ASIA‐LEMS classification, may not yield accurate results at this stage. However, the H‐reflex, measurable through neurophysiological methods, offers a potential solution to overcome the challenge of obtaining consistent results in the early stages, even in cases where patient cooperation is limited.^16^ Therefore, the H‐reflex may be more sensitive in detecting early subclinical changes that may not be evident through clinical evaluation alone.^59^

The findings in the present research highlight that the sooner SCI patients are transferred to a rehabilitation facility, the higher the probability of complete bladder recovery. This aligns with evidence from previous studies emphasizing the advantages of early transfer to rehabilitation facilities for SCI patients.^60^, ^61^ For example, Herzer et al.^62^ conducted a comprehensive analysis of data from 3937 traumatic SCI patients in the United States from 2000 to 2014, revealing that early transfer to rehabilitation facilities enhances patients' functional status upon discharge. Similarly, Scivoletto et al.^63^ studied 150 patients from an Italian rehabilitation hospital, observing that individuals admitted to rehabilitation within 1 month of injury exhibited significantly higher scores in terms of the MBI and activity at the time of discharge compared to those admitted later. Likewise, Sumida et al.^60^ investigated 123 patients with traumatic SCI from 17 institutions in Japan, finding that early transfer to rehabilitation facilities led to improved physical functional independence measured by the ASIA motor score. In academic settings, it is customary for individuals with SCI to receive initial treatment in acute care settings before being transferred to rehabilitation facilities once their condition stabilizes.^28^, ^60^ The timing of this transition is influenced by clinical variables such as the need for surgical intervention or intensive care. Notably, patients with more severe disease or injury, leading to greater challenges in bladder emptying, may require longer stays in acute care.^64^, ^65^ Extended periods spent in acute care with limited mobility unfortunately increase the likelihood of complications.^27^ Moreover, the risk of complications becomes even more pronounced when patients stay longer in acute care with restricted mobility.^27^ The imperative to enhance communication between acute care providers and rehabilitation professionals is crucial to reduce time spent in acute care and mitigate associated complications.^62^

Interestingly, subgroup analysis revealed that while the overall predictive power of the HALT was high (aROC = 0.878, 95% CI: 0.823–0.933), its performance further increased in patients with incomplete injuries (aROC = 0.973, 95% CI: 0.940–1.000) and decreased in those with complete injuries (aROC = 0.755, 95% CI: 0.648–0.861). It is noteworthy that approximately 62.8% of individuals diagnosed with SCI present incomplete injuries.^57^ The severity of the injury is closely linked to the recovery of bladder function. Patients admitted with incomplete injuries demonstrate more favorable advancements in bladder function, with 63.0% of them achieving complete bladder emptying. In contrast, only 25.6% of patients admitted with complete bladder injury manage to achieve complete bladder emptying. Consequently, the HALT approach may be particularly well suited for predicting outcomes in patients with incomplete injuries.

In our study, patients were treated in accordance with the Guidelines on Adult Neurogenic Lower Urinary Tract Dysfunction.^26^ Despite this, 61% of patients were unable to achieve complete bladder emptying within a 3‐month period, a group accurately identified by our predictive models. This identification holds significant value in enhancing counseling and facilitating the early implementation of personalized urological management, thereby positively impacting the quality of care and resource allocation. The treatment methods for NB mainly include non‐invasive behavioral modifications and pharmacotherapy, minimally invasive techniques such as sacral neuromodulation (SNM), and surgical interventions cystoplasty.^66^, ^67^ For patients with a significant chance of spontaneous recovery through non‐invasive methods, opting for a positive and accurate non‐invasive treatment such as CIC would be an appropriate choice. This approach avoids the need for surgical interventions, which can lead to complications and increased expenses. However, patients who find it difficult to regain normal bladder function using non‐invasive methods may derive advantages from the prompt implementation of minimally invasive or surgical interventions. Besides, since the soleus H‐reflex is elicited by stimulation of the tibial nerve, both HALT score and the soleus H‐reflex provide a direct theoretical basis for the treatment of PTNS. The diverse range of clinical manifestations complicates the determination of the suitable timing and appropriateness of surgical intervention. In this context, the utilization of the predictive models as a clinical decision support tool holds promise in preventing unwarranted allocation of resources and irreversible surgeries in patients with a heightened likelihood of achieving bladder emptying. Furthermore, both HALT score and H‐reflex will be instrumental in refining the patient selection and ensuring a balance among the various treatment groups by considering the anticipated likelihood of bladder function restoration in the design of future clinical trials regarding NB therapy or management.

The present study is subject to several limitations. First, the HALT data were derived from a relatively limited sample size obtained solely from a tertiary rehabilitation facility. To enhance the reliability of the predictive models, we plan to conduct a prospective study involving multiple centers and a larger sample size. Currently, we are in the process of seeking ethical approval and registering the study on clinical trial websites. Additionally, it should be acknowledged that certain indicators for patients were absent in this study. Despite the similarity in clinical characteristics between the included patients and those lost to follow‐up, the potential for selection bias remains. A third limitation is that we did not perform urodynamic studies to confirm bladder function. Although the HALT scoring system is an excellent prediction tool, spontaneous voiding does not necessarily indicate a healthy bladder dynamic. In many patients with spontaneous bladder emptying after SCI, abnormal storage and detrusor pressures may eventually lead to upper urinary tract damage.^57^

## 5 CONCLUSION

In the present study, we introduced a scoring system named HALT designed to predict bladder outcomes at the 3‐month mark following SCI. HALT incorporates three variables—H‐reflex, ASIA‐LEMS, and TLRF—and demonstrates remarkable precision and discriminative capability in prediction. Particularly noteworthy is the unprecedented predictive efficacy demonstrated by H‐reflex in early bladder outcomes among SCI patients. The HALT score and H‐reflex models hold great potential for seamless implementation in clinical settings, offering valuable support for patient counseling, rehabilitation goal setting, the customization of interventions, and patient stratification in the design of future clinical trials.

## AUTHOR CONTRIBUTIONS

This study was designed and managed by Xiaolong Sun and Hua Yuan. Xiangbo Wu, Xiao Xi, and Mulan Xu analyzed the data and wrote the manuscript. Material preparation, data collection, and analysis were performed by Ming Gao, Ying Liang, Miaoqiao Sun, Xu Hu, Li Mao, and Xingkai Liu. Chenguang Zhao revised the manuscript. All authors have approved the final manuscript.

## FUNDING INFORMATION

This work was supported by grants from the National Natural Science Foundation of China (82272591 and 82072534) and Medical Staff Training and Boost Project of Xijing Hospital (XJZT24LY11; XJZT24JC19; XJZT24QN20).

## CONFLICT OF INTEREST STATEMENT

The authors have no conflict of interest.

## Supporting information


Data S1.

Table S1.

Table S2.

## Data Availability

The datasets used and analyzed during the current study are available from the corresponding author upon reasonable request.
