# Supplementary material for: Prediction of early bladder outcomes after spinal cord injury: The HALT score
Source: CNS Neurosci Ther. 2024 Feb 7;30(2):e14628. doi: 10.1111/cns.14628 (PMC10850821; doi:10.1111/cns.14628)
Supplement: Supplementary file 2 — Table S1. [file CNS-30-e14628-s001.docx]

| **SUPPLEMENTAL TABLE 1** Baseline characteristics of patients included in the analysis and lost to follow-up | | | |
| --- | --- | --- | --- |
| **Variable** | **Study sample**  (n=202) | **Lost at follow up** (n=21) | ***P-value*** |
| ***Demographic characteristics*** |  |  |  |
| **Age, mean ±SD, years** | 40.15±12.48 | 44.52±15.53 | 0.137 (Student's t-test) |
| **Gender** |  |  | 0.579 (χ^2^) |
| Male | 159 (78.7%) | 15 (71.4%) |  |
| Female | 43 (21.3%) | 6 (28.6%) |  |
| ***Clinical parameters*** |  |  |  |
| **Surgery** |  |  | 0.111 (χ^2^) |
| Yes | 192 (95.0%) | 18 (85.7%) |  |
| No | 10 (5.0%) | 3 (14.3%) |  |
| **Etiology** |  |  | 0.068 (χ^2^) |
| Non-traumatic | 21 (10.4%) | 5 (23.8%) |  |
| Traumatic | 181 (89.6%) | 16 (76.2%) |  |
| **TLRF, median (IQR), days** | 21.0 (10.0, 35.0) | 18.0 (11.0, 31.5) | 0.802 (Mann–Whitney U) |
| ***Neurological status*** |  |  |  |
| **Neurological Level** |  |  | 1.000 (χ^2^) |
| C1-L5 | 193 (95.5%) | 21 (100.0%) |  |
| S1-S5 | 9 (4.5%) | 0 (0.0%) |  |
| **Severity of initial neurological deficit** |  |  | 0.195 (χ^2^) |
| AIS grade A | 129 (63.9%) | 12 (57.1%) |  |
| AIS grade B | 30 (14.9%) | 1 (4.8%) |  |
| AIS grade C | 22 (10.9%) | 3 (14.3%) |  |
| AIS grade D | 21 (10.4%) | 5 (23.8%) |  |
| AIS grade E | 0 (0.0%) | 0 (0.0%) |  |
| **ASIA-LEM****S, median (IQR)** | 0 (0, 20.0) | 0 (0, 24.0) | 0.768 (Mann–Whitney U) |
| **S4-5 dermatome sensation** |  |  | 0.246 (χ^2^) |
| Normal | 85 (42.1%) | 5 (23.8%) |  |
| Impaired | 42 (20.8%) | 5 (23.8%) |  |
| Absent | 75 (37.1%) | 11 (52.4%) |  |
| **Deep anal pressure** |  |  | 0.297 (χ^2^) |
| Present | 120 (59.4%) | 10 (47.6%) |  |
| Absent | 82 (40.6%) | 11 (52.4%) |  |
| **Anal reflex** |  |  | 0.277 (χ^2^) |
| Present | 121 (59.9%) | 10 (47.6%) |  |
| Absent | 81 (40.1%) | 11 (52.4%) |  |
| **Voluntary anal contraction** |  |  | 0.074 (χ^2^) |
| Present | 118 (54.4%) | 8 (38.1%) |  |
| Absent | 84 (41.6%) | 13 (61.9%) |  |
| ***Functional status*** |  |  |  |
| **MBI, median (IQR)** | 11.0 (5.0, 26.0) | 7.0 (2.5, 14.5) | 0.174 (Mann–Whitney U) |
| **SCIM total score, median (IQR)** | 17.0 (10.0, 28.0) | 12.0 (8.0, 22.0) | 0.071 (Mann–Whitney U) |
| ***Complications*** |  |  |  |
| **Urinary tract infection** |  |  | 0.779 (χ^2^) |
| Yes | 52 (25.7%) | 6 (28.6%) |  |
| No | 150 (74.3%) | 15 (71.4%) |  |
| ***Electrophysiology*** |  |  |  |
| **H-reflex** |  |  | 0.160 (χ^2^) |
| H-reflex (-) | 113 (55.9%) | 8 (38.1%) |  |
| H-reflex (±) | 25 (12.4%) | 2 (9.5%) |  |
| H-reflex (+) | 64 (31.7%) | 11 (52.4%) |  |
| SD, Standard Deviation; IQR, interquartile range; TLRF, time from lesion to rehabilitation facility; AIS, American Spinal Injury Association Impairment Scale; ASIA-LEMS, the American Spinal Injury Association lower extremity motor score; MBI, the Modified Barthel Index; SCIM, the Spinal Cord Independence Measure | | | |
