# Supplementary material for: Prediction of early bladder outcomes after spinal cord injury: The HALT score
Source: CNS Neurosci Ther. 2024 Feb 7;30(2):e14628. doi: 10.1111/cns.14628 (PMC10850821; doi:10.1111/cns.14628)
Supplement: Supplementary file 3 — Table S2. [file CNS-30-e14628-s002.docx]

| **SUPPLEMENTAL TABLE 2** Estimated probabilities of complete bladder emptying at 3-month after SCI based on total score within 40 days | |
| --- | --- |
| **Score** | **Estimated Probabilities** |
| 0 | 0.040 |
| 1 | 0.055 |
| 2 | 0.074 |
| 3 | 0.099 |
| 4 | 0.131 |
| 5 | 0.172 |
| 6 | 0.222 |
| 7 | 0.283 |
| 8 | 0.352 |
| 9 | 0.428 |
| 10 | 0.507 |
| 11 | 0.586 |
| 12 | 0.661 |
| 13 | 0.729 |
| 14 | 0.787 |
| 15 | 0.836 |
| 16 | 0.875 |
| 17 | 0.906 |
| 18 | 0.930 |
| 19 | 0.948 |
| 20 | 0.962 |
| 21 | 0.972 |
| 22 | 0.980 |
| 23 | 0.985 |
| 24 | 0.989 |
